# Exploring the feasibility of Conjoint Analysis as a tool for prioritizing innovations for implementation

**DOI:** 10.1186/1748-5908-8-56

**Published:** 2013-05-29

**Authors:** Katherine Farley, Carl Thompson, Andria Hanbury, Duncan Chambers

**Affiliations:** 1 Department of Health Sciences, The University of York, York YO10 5DD, England; 2 Centre for Reviews and Dissemination, The University of York, York YO10 5DD, England

**Keywords:** Conjoint Analysis, Healthcare, Innovation, Decision-making, Implementation, Preference

## Abstract

**Background:**

In an era of scarce and competing priorities for implementation, choosing what to implement is a key decision point for many behavioural change projects. The values and attitudes of the professionals and managers involved inevitably impact the priority attached to decision options. Reliably capturing such values is challenging.

**Methods:**

This paper presents an approach for capturing and incorporating professional values into the prioritization of healthcare innovations being considered for adoption. Conjoint Analysis (CA) was used in a single UK Primary Care Trust to measure the priorities of healthcare professionals working with women with postnatal depression. Rating-based CA data was gathered using a questionnaire and then mapped onto 12 interventions being considered as a means of improving the management of postnatal depression.

**Results:**

The ‘impact on patient care’ and the ‘quality of supporting evidence’ associated with the potential innovations were the most influential in shaping priorities. Professionals were least influenced by whether an innovation was an existing national or local priority, or whether current practice in the Trust was meeting minimum standards. Ranking the 12 innovations by the preferences of potential adopters revealed ‘guided self help’ was the top priority for implementation and ‘screening questions for post natal depression’ the least. When other factors were considered (such as the presence of routine data or planned implementation activity elsewhere in the Trust), the project team chose to combine the eight related treatments and implement these as a single innovation referred to as ‘psychological therapies’.

**Conclusions:**

Using Conjoint Analysis to prioritise potential innovation implementation options is a feasible means of capturing the utility of stakeholders and thus increasing the chances of an innovation being adopted. There are some practical barriers to overcome such as increasing response rates to conjoint surveys before routine and unevaluated use of this technique should be considered.

## Background

Innovation implementation (the process of integrating research findings into behaviours at the level of adopters) happens ‘in context’. Context in many healthcare systems includes scarce (or at least finite) resources, variability in adoption of existing innovations, and ways of changing behaviour that often incur their own costs but are rarely factored into the final estimate of the cost effectiveness of innovation adoption [1,2].

One key element of current implementation context is the growing volume of innovation that health system decision and policy makers are compelled to consider for implementation. By way of example, General Practitioners in the UK face up to 30 new pieces of guidance per month, far more than can feasibly be adopted by a multidisciplinary team, practice or clinic [3]. Faced with scarce resources and increasing demand [4], systems must prioritize and decide which innovations to implement. Guidance on how prioritization should be undertaken by potential adopters is scarce.

Policy makers have resorted to economic criteria, such as cost-effectiveness, to help decide which innovations should be available for services to consider. However, ranking innovations on the basis of economic attributes such as cost-effectiveness employed by NICE or program budgeting and marginal analysis [5] misses the role that other factors play in the choice to adopt or not at service purchaser or provider levels [4,6,7].

Influential theories of innovation diffusion generally [7] and healthcare specifically [4,6] suggest multiple general determinants of adoption behaviour. Some theorists [6] identify a large number of possible determinants: the characteristics of the innovation; system antecedents (structure of the organisation, absorptive capacity for new knowledge, and receptive context for change); system readiness; characteristics of adopters; communication and influence and a wider ‘outer’ context of politics and structures. Other models adopt a more parsimonious approach that focuses on the characteristics of innovations and prior conditions [7]. The attitudes and values held by potential adopters exist as variables in almost all theoretical models of innovation adoption and are a focus within the field of implementation science [8,9].

Clearly, attitudes are influenced by many factors and are unlikely to be decisive in themselves in determining whether an innovation is adopted or behaviour changed. Indeed, some evidence indicates that, in specific healthcare contexts, compatibility of guideline attributes with clinicians’ values can be negatively related to desired behaviour change. Foy et al. tested the influence of attributes of clinical recommendations on compliance with recommendations using a pre- and post-intervention research design [10]. Foy et al. found that while guideline-norm ‘fit’ and guideline compliance before and after a behaviour change intervention (audit and feedback) were positively related, guideline recommendations seen as incompatible with clinician norms showed greater change following the intervention [10]. Foy and colleagues’ pre-post design was limited by the absence of data points within the intervention period, thus the effects of the intervention on compliance behaviour during the intervention period are unknown [10]. Further, since Foy et al. used a qualitative approach to establishing innovation attributes (*i*.*e*., using focus groups and interviews), it is possible that different experts might have described different attributes.

We sought to use clinicians’ values in a slightly different way. Rather than evaluating their relationship with the effect of the behaviour change intervention, we built them into the selection of an innovation to be implemented. This study forms the initial phase in developing a targeted implementation strategy. In the subsequent phase, we examine six other determinants in order to tailor a multi-faceted implementation strategy to the barriers associated with each factor [11]. Our rationale was a simple one: mindfulness of a ‘fit’ between a potential innovation’s characteristics and potential adopter values and norms would lead to focusing on an innovation for implementation that has (at least) a ‘fighting chance’ of adoption. Omitting to address the value clinicians assign to the attributes of an innovation theoretically lowers the chances of the innovation being adopted [7].

One technique for understanding the value of a product’s attributes is Conjoint Analysis. CA is a stated preference method with its roots in mathematical psychology [12,13] and Lancaster’s theory of value [14]. Respondents are asked to choose between hypothetical products (or innovations) with different levels of a limited number of attributes. The findings can then be used to rank competing innovations whose levels of these attributes have been scored in advance. CA makes two assumptions: that innovations can be described according to their attributes, and that the value of an innovation (to an individual) is a product of these collective attributes [15]. It has the advantage of simultaneously estimating the value/utility placed on a product/service (or innovation) while also identifying the relative (to other attributes) importance of the attributes making up the innovation. CA also describes the extent to which individuals are willing to trade off one attribute to gain another (for example, cost vs. quality). It has been applied to many areas: market research [16], private sector environmental and transport economics, public service redesign and planning [17]. Specific health applications include in vitro fertilization [18], orthodontic services [14,19], and liver transplantation [20]. CA has not been widely used in implementation research and to the best of our knowledge has never been used to prioritize possible targets for implementation efforts.

To demonstrate how this technique can be successfully applied to the prioritization of healthcare innovations, we present an application of Conjoint Analysis to the implementation of innovations for women with postnatal depression in one UK NHS Primary Care Trust (PCT). The results are not intended to be generalizable to other settings; they are presented here to illustrate the application of CA to a key stage in the implementation process and the challenges involved.

## Methods

### Conjoint Analysis

The start point for the project was the selection of 1 (of a potential 12) recommended innovations for mild to moderate postnatal depression competing for implementation resources in the PCT. We followed the five-stage structure of a typical Conjoint Analysis project [outlined in Table 1] adapted from Ryan et al. [15]. In addition to the five stages of a conjoint study, two further stages were included to apply the technique to the real healthcare innovations: scoring of potential innovations (treatments) to be prioritized, and matching of stakeholder preferences to these innovations.

**Table 1:** Conjoint Analysis process

**Conjoint Analysis requirements**	**Corresponding stage in process**
Conjoint Analysis relies on the development of a set of attributes or criteria that describe a given product.	
	Stage 1: Identifying plausible and meaningful innovation attributes that could be used to characterize healthcare innovations (for example, financial cost).
Levels of each attribute (such as £0, £100, £1,000) for each criterion are assigned. These need to be meaningful and able to be traded off.	Stage 2: Operationalizing the attributes of innovations and their levels. Twelve postnatal depression treatments being considered by the Trust for implementation were described using these attributes.
Hypothetical scenarios with different combinations of attribute levels are included in the questionnaire.	Stage 3: A fractional factorial design is used to identify the number of hypothetical scenarios to be included.
Eliciting stakeholder preferences. In choosing or rating, respondents must trade off some elements of the innovation (for example, cost) for an increase in another attribute (for example, quality); a process known as the ‘marginal rate of substitution,’ [12]. Analysis of the choices made yields estimates of how much respondent stakeholders are prepared to trade off in their preferred attributes in order to receive their preferred combination of attributes.	Stage 4: Information about clinician preferences for innovation attributes is collected using a questionnaire. Respondents rate hypothetical (but feasible) innovations, products or services described using these criteria. Analysis reveals the importance of each attribute, and the clinician preferences for each attribute at each of its component levels. Alternative methods such as Choice Based Conjoint are available that quantify individuals’ values in terms of their willingness to pay (WTP) for an innovation, but rating scales have been shown to perform well in eliciting preferences for healthcare services [27].
Estimating utilities to determine the importance of each attribute to stakeholders.	Stage 5: Analysis of data using Sawtooth software provides preference scores (utilities) for each attribute.
Independent scoring of innovations.	Stage 6: ‘Scoring’ of potential postnatal depression treatments using the attributes and levels identified.
Matching clinician preferences for innovations with the scored innovations	Stage 7: The 12 innovations were ranked according to the preferences of the clinicians who would have to implement them.

#### Stage 1: Identifying innovation attributes

The selection of attributes was critical, as omitting important attributes would weaken the internal validity of the resulting conjoint design and analysis [21]. We sought to balance using enough attributes to efficiently describe a wide range of possible innovations with the need for the eventual mix to have enough face validity to be meaningful to both clinicians and policy makers. Attributes chosen were those that were, first, considered important by policy makers currently prioritizing resources to support implementation from the Primary Care Trust; second, described by Rogers’ theoretical model as ‘perceived attributes’ [7]; and third, that were theoretically identified barriers to, and facilitators of, change [4].

Selection of attributes was also guided by criteria identified by Grimshaw et al.: the local burden of disease, the availability of ‘effective and efficient healthcare interventions,’ and ‘local evidence of current suboptimal performance’ [4]. Because our conjoint exercise was rooted in a specific local attempt to change behaviour, we also considered the influence of having routine data on clinical behaviour available and the policy makers’ need to apply the ascertained preferences to a diverse range of healthcare innovations in the future. The final list of attributes is shown in Table 2. One of these attributes was the cost of the innovation, the inclusion of which makes visible the trade-offs made by clinicians between having more of their preferred attributes and paying more in resources.

**Table 2:** Attributes and levels

**Characteristic**	**Level**
Impact on care	Significant improvement
	Moderate
	Limited
Costs	Low
	Moderate
	High
Local health needs	Low prevalence
	High prevalence
Minimum standards	No, not meeting minimum standards.
	Yes, meeting minimum standards.
Strength of supporting evidence	No supporting evidence.
	Limited supporting evidence.
	Moderate supporting evidence.
	Strong supporting evidence.
Priority	National priority.
	Local priority.
	Both local and national priority.
Existence of local expertise	No, there is no local expertise.
	Yes, there is local expertise.
Constant	

Selection of attributes and the language used to express them influences the validity of responses [22]. As well as affecting response rates, using attributes that adequately describe a wide range of types of innovations improves external validity and generalizability of utilities to new innovations. To test internal validity, the questionnaire included five ‘hold out’ questions [23,24]. These hold out tasks were not included in the calculation of utilities; they were used only to compare modelled and predicted choices against choices already made [24]. Finally, face validity is tested using qualitative methods, making it possible to improve attribute selection and questionnaire design [25]. We gathered informal qualitative feedback from clinicians in similar roles as our study population to improve our CA questionnaire.

#### Stage 2: Operationalizing the attributes of innovations and their levels

Once seven attributes were defined, the component levels were identified (Table 2). For simplicity and to maximize response rates, the number of attributes and levels should be as low as possible [26]. The attributes were then described in ways that would be meaningful to stakeholders. While the combinations of attributes are used to generate hypothetical innovations, it is important that the attributes themselves have concrete and relevant descriptions [15]. Attribute levels were thus presented in a manner as close as possible to the natural units policymakers encounter. For example, cost was expressed as pounds (£) per patient, and burden of disease as prevalence (% of population affected or rate per 1,000 population members). In order to check the clarity and wording of the descriptions, identify any missing attributes and levels, and provide a general check of the questionnaire, the questionnaire was piloted with 12 GPs [27].

#### Stage 3: Identifying which scenarios to present

The SPSS orthoplan (http://www.spss.com) procedure, based on the methods for estimating orthogonal main effects plans of Addelman [28], produced a fractional factorial design (The SPSS ‘orthoplan’ procedure (http://www.spss.com) was used to generate the design) with 16 scenarios. Details of the decision rules used to reduce the design to 16 scenarios can be found at http://www.springanalytica.com/UNIZ/PhD/orthoplan.pdf p9. To ensure internal consistency, five additional scenarios were included that, rationally, should produce a higher score than others.

#### Stage 4: Eliciting stakeholder preference using Conjoint Analysis

Data collection took the form of a rating-based questionnaire. Participants were presented with 16 hypothetical innovations, each described by its attributes and varying levels (for example, innovation A is high cost, has a strong evidence base, with significant variations in local practice; innovation B is low cost, has a weaker evidence base, and less variation in local practice). To indicate the likelihood of prioritization, participants were asked to rate each of the series of 16 hypothetical innovations on a seven point Likert-type scale anchored at ‘Very likely to prioritize this guideline’ through to ‘Very unlikely to prioritize this guideline’.

The questionnaire was delivered to 1,200 healthcare professionals involved in the care of women with post natal depression (GPs, health visitors, and nurse practitioners). As part of a separate trial comparing postal and electronic delivery processes, half of the sample was randomly allocated to receive postal questionnaires at their work address and the other half a personalized email with a hyperlink to an online version. The questionnaire was endorsed by the Trust’s Medical Director and piloted to check that the attributes, language and format were appropriate. Following best practice guidance on increasing response rates [29,30], two reminders were sent to recipients; the first, after two weeks containing a short text reminding the recipient about the questionnaire. The second was sent three weeks later containing a second copy of the questionnaire or link to the online version. The questionnaire was also promoted by telephone by members of the research team in their capacity as internal Trust auditors and trainers.

#### Stage 5: Analysis - extracting utilities

Responses were analysed using Sawtooth software’s Conjoint Value Analysis (CVA module) (http://www.sawtooth.com). The analysis is an ordinary least squares regression (OLS) in which the dependent variable is the priority associated with an innovation (treated as interval data), and the independent variables are the innovation attributes at their component levels (dummy coded where necessary). The analysis produces utilities (preference values) for each attribute and for each level of the attributes. We chose the simple OLS method of generating utilities from the data, as OLS is suitable for the ratings-based data we collected, and also that others wishing to use the technique could do using easily available statistical software (*e*.*g*., Excel, Stata or SPSS).

#### Stage 6: ‘Scoring’ of potential innovations for implementation

The Primary Care Trust provided a list of 12 postnatal depression innovations being considered for implementation. For each innovation, we used resources such as systematic reviews, NICE guidelines, and local data provided by the Trust to ‘score’ innovations against the attribute criteria; *i*.*e*., according to their cost, quality of their evidence base, levels of variation in practice, etc. Scoring of the attributes of our ‘real’ postnatal depression innovations was subjective. The value of using Conjoint Analysis in this way is the ability to adjust attribute values to assess the potential impact upon prioritization – essentially, being able to ask, ‘What if [the innovation had different attribute values]?’

#### Stage 7: Matching preferences with scored innovations

In this final stage, the preferences of clinicians were matched to the 12 postnatal depression innovations. In order to unite preferences (from Stage 5) and the attributes of the innovations (from Stage 6), the utilities for each attribute at various levels are summed for each individual respondent. The option with the highest mean utility in the sample of clinicians represents the ‘first choice’ and so the most favoured.

## Results

The questionnaire was completed by 11% (N = 139) of the sample, with an equal number of responses from postal and email delivery methods.

### Stakeholder attribute priorities

The analysis firstly revealed those attributes most strongly influencing clinicians’ prioritisation decisions. Table 3 indicates that the attributes with the greatest impact on clinicians’ ratings were the ‘impact on patient care’ and the ‘quality of supporting evidence.’ Stakeholders were least influenced by whether an innovation was a national or local priority, or whether current practice was meeting minimum standards. The internal validity and real-world applicability of the survey was confirmed as respondents appear to have responded rationally: ‘Low costs’ were scored as preferable to ‘high costs’, and ‘significant impact on care’ scored as preferable to ‘limited impact on care’. Robustness was further supported with reference to the hold out cases, which were accurately predicted in the model with a coefficient of determination (correlation coefficient squared) of 93% (Pearson’s r = .96, p < 0.001). This figure indicates the proportion of the expected preference explained by the actual preference [30].

**Table 3:** **Stakeholders**’ **prioritization of characteristics**

**Characteristic**	**Ranking from electronic survey**	**Ranking from paper survey**
Impact on care	1	2
Strength of supporting evidence	2	1
Local health needs of patients/clients	3	3
Costs associated with new ways of working	4	4
Local expertise	5	5
Meeting minimum standards	6	6
National or local priority	7	7

### Linking priorities with innovation attributes

Each innovation was independently assessed using the seven attributes. Table 4 shows the attributes assigned to each innovation. The reader will note that three of the attributes are (necessarily) ‘fixed’ in our illustrative example: ‘local health needs,’ ‘local expertise,’ ‘minimum standards.’ The technique’s application here is to a problem in which policy makers faced competing priorities for innovation investment/adoption within a single clinical domain: post natal depression. Hence ‘need,’ ‘local expertise’ [in managing PND], and ‘standards of care’ [associated with PND] did not vary. In other applications, the priorities for innovation adoption will cross clinical domains or problem areas and so attributes will vary.

**Table 4:** Independent assessment of postnatal depression innovations (allocation of criteria)

**Innovation**	**Impact on care**	**Strength of evidence**	**Local health needs**	**Costs**	**Local expertise**	**Min. standards**	**Priority**
NICE diagnostic questions	Limited	No evidence	High	Low	Yes	No	Local
Guided self help	Moderate	Moderate	High	Low	Yes	No	National and local
Computerised CBT	Limited	Moderate	High	Low	Yes	No	National and local
Exercise	Limited	Limited	High	Moderate	Yes	No	Local
Health visitor listening visits	Moderate	Moderate	High	Moderate	Yes	No	National and local
Practice counsellor referral	Moderate	Moderate	High	High	Yes	No	National and local
Brief CBT	Limited	Limited	High	Moderate	Yes	No	National and local
Full CBT	Moderate	Limited	High	High	Yes	No	National and local
Anti- depressants	Limited	Limited	High	Low	Yes	No	Local
Anti-depressants and psychological therapies together	Limited	Moderate	High	Low	Yes	No	Local
EPDS diagnostic tool	Moderate	Limited	High	Low	Yes	No	Local

After ranking the 12 innovations according to the preferences for their attributes (Table 5), ‘guided self help’ was the top priority for implementation and ‘screening questions for post natal depression’ the least. The ‘diagnostic tool’ was the innovation that was ranked first solely on preferences. However, the team and PCT end user of the analysis had also to consider factors important in the NHS health economy served by the project. These included application to adequate service-user numbers, the number of clinicians associated with the interventions, other related activity within the Trust, data availability, the recording process, and commissioning patterns locally. We did not include these factors in the design for two reasons. Firstly, in conjoint designs, the number of attributes affects the number of scenarios included in the survey as well as the required sample size. Including more than seven attributes would have required a far larger number of scenarios given the sample size. Secondly, these were pragmatic factors that were difficult to categorize in levels suitable for conjoint designs. Once these considerations were added to the information on preferences alone, the study team chose to implement a combination of eight psychological therapy interventions.

**Table 5:** Ranking of postnatal depression innovations

**Innovation**	**Rank from postal**	**Rank from electronic**
Guided self help	1	1
HV listening visit	2	2
EPDS	3	4
Practice counsellor	4	3
Full CBT	5	6
Computerised CBT	6	5
Anti-depressants and psychological therapy	7	7
Anti-depressants	8	9
Brief CBT	9	8
Exercise	10	10
NICE screening questions	11	11

### Utilities of attributes

Table 6 provides the utilities for each attribute at each of their levels. Stage 5 produced a ranked list of the potential innovations. Thus, the final rank reflects the value of the innovation for the clinician sample given the attributes of the innovations.

**Table 6:** Utilities of innovation characteristics

**Characteristic**	**Level**	**Utility estimate**	**Std. error**	**Innovation A**	**Innovation B**
**Impact on care**	Significant improvement	-0.228	0.111	√	√
	Moderate	0.078	0.130		
	Limited	0.150	0.130		
Costs	Low	0.252	0.111		√
	Moderate	-0.090	0.130	√	
	High	-0.162	0.130		
Local health needs	Low prevalence	-0.207	0.083		
	High prevalence	0.207	0.083	√	√
Minimum standards	No, not meeting minimum standards.	-0.324	0.083		√
	Yes, meeting minimum standards.	0.324	0.083	√	
Strength of supporting evidence	No supporting evidence.	-0.243	0.144		
	Limited supporting evidence.	-0.135	0.144		
	Moderate supporting evidence.	-0.027	0.144		√
	Strong supporting evidence.	0.405s	0.144	√	
Priority	National priority	-0.108	0.111	√	
	Local priority	-0.144	0.130		√
	Both local and national priority	0.252	0.130		
Existence of local expertise	No, there is no local expertise.	0.018	0.083	√	
	Yes, there is local expertise.	-0.018	0.083		√
Constant		0.048	0.096		

Consider Table 6, which contains two example hypothetical innovations that we shall call, ‘Innovation A’ and ‘Innovation B.’ We can see that hypothetical ‘Innovation A’ has a significant impact on care, with strong supporting evidence, is able to be delivered at moderate cost, applies to patients with a high prevalence condition, is a national priority, has no local expertise in place, and is already meeting minimum standards. Thus, innovation A has a total utility of:

Constant + utility for attribute A + utility for attribute B + utility for attribute *k* Or 0.05 + −.023 + −0.09 + 0.21 + 0.32 + 0.40 +0.25 + 0.02 = 0.94

In contrast, ‘Innovation B’ has a significant impact on care, has only moderate supporting evidence, but is deliverable at low cost, to a high prevalence patient group, local expertise is present, is a recognized local priority, and minimum standards are not being met. Innovation B would have a total utility of −0.15. Thus, innovation A is preferred over innovation B. Examining the total utilities for the actual postnatal depression innovations enabled us to produce a list of the 12 innovations (Table 5) ranked by the predicted utility (value) that the respondents would get from the innovation. Of these 12 innovations, we selected 8 related innovations which we combined to make one single innovation: ‘psychological therapies,’ the aim being to increase the adoption of any one of these therapies.

## Discussion

Using the results of a local survey conducted in a Primary Care Trust, this paper describes one solution to the challenge of incorporating clinician preferences into the prioritization of innovations in healthcare systems in which resources are finite and limited. In doing so, this study demonstrates the feasibility of the application of Conjoint Analysis to implementation. The analysis provided three things:

1. The importance of the attributes of innovations generally;

2. Their importance at various levels;

3. And a ranked picture of innovations according to the preferences of the people involved in having to implement them.

There are other widely used methods for prioritizing innovations for adoption or investment. Criteria such as multi-criteria decision analysis [31], budget impact analysis [32], and cost-consequence analysis [33] are all useful. However, the conjoint analytic approach enabled us to consider the innovation options most likely to ‘fit’ local preferences before we developed an implementation strategy that went on to measure and target other determinants of innovation adoption. Improving the ‘fit’ with local values as well as mapping and targeting the multitude of other determinants, should – in theory – increase the efficiency of the eventual implementation strategy [6]. A more efficient implementation strategy will, all things being equal, reduce the costs of behaviour change approaches and thus increase ‘policy cost effectiveness’ – *i*.*e*., an estimate of costs vs. impact that takes into account the costs of changing behaviour as well as the cost effectiveness of the innovation itself [1].

By applying Conjoint Analysis to healthcare innovation preferences for implementation, we were able to provide a visible rationale for the decision of which innovation to invest scarce time, money and human resources on. To the best of our knowledge, this is the first time CA has been used in implementation science in this way. Previous applications of CA to service improvement, design or planning have primarily been designed to inform single services (for example, [18,19]). While applying CA to single service design or improvement may be valuable, it may not be efficient. Because CA utilities relate to the attributes of products or services, CA results can also be used in the future for ‘different-but-similar’ products. Enabling organizations to reuse preferences that can be applied to a range of topics and innovations, CA could be a more efficient means of gathering stakeholder data than repeatedly surveying people.

There remain some challenges in applying CA. Perhaps the most significant is identifying effective sampling strategies and achieving high response rates. Despite adhering to evidence-based sampling strategies [34], our exercise resulted in only an 11% response rate. Conjoint Analysis questionnaires in healthcare can be complex, a complexity that is exacerbated because decomposing and describing healthcare innovations on the basis of their compound attributes is difficult. Making descriptions informative, nuanced and yet meaningful and accessible (to non-technical audiences) is difficult. Healthcare technologies and the factors involved in decision-making in this sector may be more numerous than for products in which Conjoint Analysis has been traditionally used. Higher response rates have been achieved in other healthcare contexts, suggesting that this challenge can be overcome [21,35].

A second difficulty is that the stability of stakeholder preferences over time is less well known. Although some studies in non-healthcare contexts suggest that preferences are relatively stable [36,37], there is scope for future research into the effect of time on preferences. Theoretically, once derived, preference models could be applied to future innovation implementation choices. More work is required to establish the stability of preferences in this context.

Balancing conciseness of language in the questionnaire and meaningfulness to clinicians was challenging. A further challenge was identifying attributes and levels that could be applied to a diverse range of innovations; for example, diagnostic techniques and treatment modalities.

While ratings-based conjoint was preferred in our context [34], rating questionnaires can be difficult to complete. More technically, individual-level utilities are not available in ratings approaches. A variant of CA, Discrete Choice Experimentation (DCE), offers an alternative built around random utility theory (RUT). DCE is widely used in health economics [25]. In contrast to rating-based Conjoint Analysis, respondents are faced with direct choices between options (‘Of these two options which would you choose?’), an approach that mirrors real decision making.

Finally, the CA approach outlined in this paper applied only to the adoption of innovations into a healthcare system. The approach tells us nothing about the choices people make to stop using or dis-adopt an intervention. There is considerable scope for adapting the approach to examining the relationship between value and norm compatibility and behaviour that is not desired by those seeking to foster sustainable adoption.

## Conclusions

Increasingly, healthcare systems are faced with the problem of which innovations to implement. Conventional methods of prioritization are often intuitive, opaque, and based on socio-political factors such as which stakeholder group voice carries the most weight. There are other determinants of course, but the probability of innovations being adopted is influenced – if only in part – by the values and preferences of professionals (potential adopters) in healthcare systems and the characteristics of innovations. However, preferences can be difficult to gather and analyze systematically, rigorously, reliably and efficiently. Conjoint Analysis, with its central premise that an innovation’s value is the sum of its components, holds considerable promise. This paper has shown that despite the practical hurdles to be overcome, the proof of principle exists: preferences can be mapped, matched to innovation characteristics, and used to shape the design and implementation of interventions to change behaviour and encourage adoption.

### Ethical approval

Ethical approval was granted for this study by National Research Ethics Service (Reference 09/H1311/81).

## Abbreviations

CA: Conjoint Analysis; CCG: Clinical Commissioning Group; DCE: Discrete Choice Experiments; DoH: Department of Health; GP: General Practitioner; NICE: National Institute for Health and Clinical Excellence; PCT: Primary Care Trust; RUT: Random Utility Theory; TRiP-LaB: Translating Research into Practice in Leeds and Bradford.

## Competing interests

The authors declare that they have no competing interests.

## Authors’ contributions

The method was identified and developed by CT, AH, KF, and DC. Design, data acquisition and analysis was conducted by CT, KF and AH. All of the authors contributed to the development and completion of the manuscript. All authors read and approved the final manuscript.
